# Synthetic Routes and Biological Evaluation of Largazole and Its Analogues as Potent Histone Deacetylase Inhibitors

**DOI:** 10.3390/molecules16064681

**Published:** 2011-06-07

**Authors:** Shang Li, Hequan Yao, Jinyi Xu, Sheng Jiang

**Affiliations:** 1 School of Pharmacy, China Pharmaceutical University, Nanjing, Jiangsu 210009, China; Tel.: +86 25 83302827; Fax: +86 25 83271445; 2 Shanghai Institute of Technology, Shanghai 210032, China; 3 Laboratory of Peptide Chemistry, Guangzhou Institute of Biomedicine and Health, CAS, Guangzhou, Guangdong 510530, China; Tel.: +86 18688888237; Fax: +86 20 32015299

**Keywords:** largazole, histone deacetylase inhibitor, natural products, total synthesis, biological evaluation

## Abstract

Natural products with interesting biological properties and structural diversity have often served as valuable lead drug candidates for the treatment of various human diseases. Largazole, isolated from the marine cyanobacterium *Symploca* sp. has exhibited potent inhibitory activity against many cancer cell lines. Besides, it shows remarkable selectivity between transformed and nontransformed cells, which is the main disadvantage of other antitumor natural products such as paclitaxel and actinomycin D. Due to its potential as a potent and selective anticancer drug candidate, a great deal of attention has been focused on largazole and its analogues. It is the aim of this review to highlight synthetic aspects of largazole and its analogues as well as their preliminary structure–activity relationship studies.

## 1. Introduction

**Figure 1:**
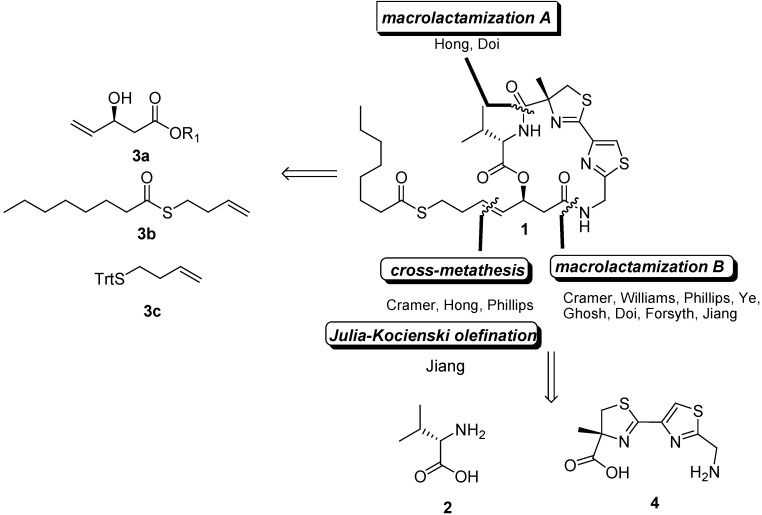
Synthesis strategies and key building fragments.

Chromatin template activities, including DNA transcription, replication, and repair, are regulated by a variety of posttranslational modifications, among which histone acetylation plays a prominent role. Histone deacetylase inhibitors (HDACIs) have long been used in psychiatry and neurology as mood stabilizers and antiepileptics. More recently, HDACIs are being studied as targeted therapies for the treatment of neurodegenerative diseases [1]. Largazole (**1**, Figure 1) is a natural macrocyclic depsipeptide isolated from a Floridian marine cyanobacterium *Symploca* sp. by Luesch and co-workers [2]. The growth-inhibitory activity of largazole is shown considerably higher for cancer cell lines (GI_50_ = 7.7 nm) than for the corresponding nontransformed cells (GI_50_ = 122 nm). It has shown promising selective biological activity for differential growth inhibition in a number of transformed and nontransformed human and murine derived cell lines. The remarkable selectivity of largazole against cancer cells has prompted research on its mode of action and its importance as a potential cancer chemotherapeutic agent, and several research groups have completed its total synthesis. This review focuses on recently developed novel synthetic routes and their applications in the development of conformational constrained analogues of largazole, as well as biological evaluation and the preliminary structure–activity relationships of largazole and its analogues are also discussed.

## 2. Synthetic Routes and Biological Evaluation of Largazole and Its Analogues

Largazole is a densely functionalized macrocyclic depsipeptide consisting of an α-methylcysteine-derived thiazoline coupled to a thiazole embedded within a 16-member macrocycle and a caprylic acid-derived thioester, which is rarely found in natural products. The retrosynthetic analysis of largazole shows that two cyclization sites A and B exist in its structure (Figure 1). To synthesize largazole, most research groups have used the following three key building blocks: valine (**2**), β-hydroxy ester **3a**, and thiazolylthiazoline **4**.

**Scheme 1:**
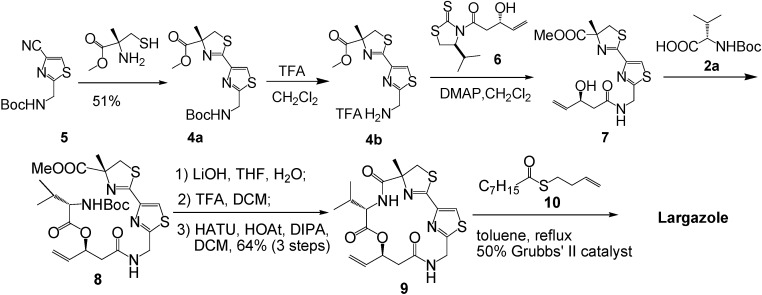
Synthetic route of largazole developed by Luesch and co-workers in collaboration with Hong and co-workers.

Luesch and co-workers were not only the first to isolate the natural largazole, but also the first to complete the total synthesis of largazole in collaboration with Hong and co-workers [3]. The condensation of compound **5** with (*R*)-2-methylcysteine methyl ester provided the key building block **4a**. Removal of the Boc group, followed by coupling of amine **4b** with **6**, which was prepared by a Nagao aldol reaction [4,5], Yamaguchi esterification or DCC-coupling reaction, with *N*-Boc-L-valine (**2a**) provided **8**. Subsequent hydrolysis and deprotection provided a precursor to the 16-member cyclic depsipeptide core. The macrocyclization of the precursor using HATU–HOAt (HATU, HOAt, *i*-Pr_2_NEt) proceeded smoothly to give **9** in 64% yield (in three steps). Thioester **10** was prepared by coupling the thioacid with 4-bromo-1-butene. The olefin cross-metathesis reaction of thioester **10** with the macrocycle **9** in the presence of 50 mol% of Grubbs’ second-generation catalyst in refluxing toluene provided largazole in 41% yield, a better result compared with that obtained in the presence of Hoveyda-generation catalyst (Scheme 1).

**Scheme 2:**
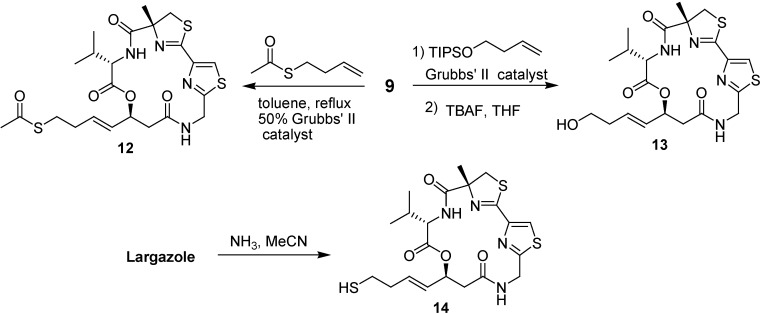
Synthesis of largazole analogues **12–14**.

By using this strategy, Luesch and his collaborators also synthesized several key analogues [6]. The acetyl analogue **12** was prepared by the olefin cross-metathesis reaction of the macrocycle **9** with thioacetic acid *S*-but-3-enyl ester (50 mol % of Grubbs’ second-generation catalyst, toluene, reflux, 50%). The same procedure was used to synthesize **13**, except 1-triisopropylsiyloxyl-3-butene was used instead of thioacetic acid *S*-but-3-enyl ester, followed by deprotection. In addition, the aminolysis of **1** provided the thiol analogue **14** in 70%–80% yield (Scheme 2).

**Scheme 3:**
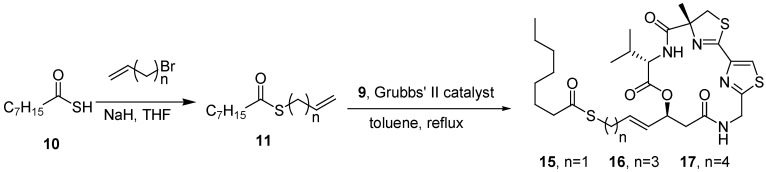
Synthesis of largazole analogues **15–17**.

The olefin cross-metathesis reaction of the macrocycle **9** with the products obtained from coupling the thioester with 3-bromo-1-propene, 5-bromo-1-pentene, and 6-bromo-1-hexene in the presence of Grubbs’ second-generation catalyst provided **15**, **16**, and **17**, respectively (Scheme 3).

**Scheme 4:**
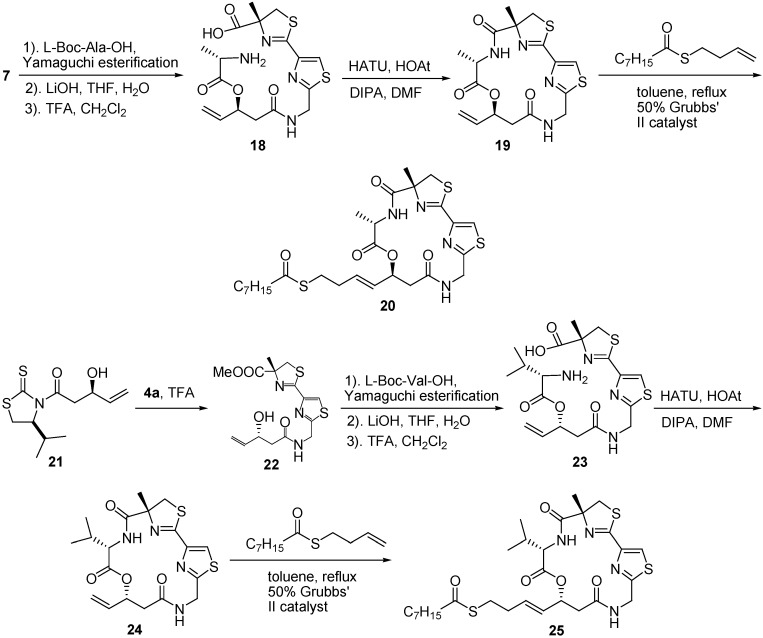
Synthesis of largazole analogues **20** and **25**.

Alanine analogue **20** and C17-epimer **25** were prepared by replacing the valine and (3*S*)-hydroxy-carboxylic acid with the alanine and (3*R*)-hydroxycarboxylic acid, respectively (Scheme 4).

Luesch and co-workers showed that histone deacetylase (HDAC) is the cellular target for largazole’s antiproliferative activity. Largazole is a prodrug, and **14** is the reactive metabolite. Largazole, its thiol analogue **14**, and its acetyl analogue **12** all exhibited similar cellular and antiproliferative activities against HDACs. However, the hydroxyl analogue **13** and the macrocycle **9** did not show any inhibitory activity. This result suggests that the thiol group is indispensable for both activities. Alanine analogue **20** showed approximately a 2- to 3-fold decrease in both activities compared with **1**. The five- and six-atom linkers in **16** and **17**, respectively, reduced the cell growth and HDAC inhibitory activity by several orders of magnitude, whereas **15** with the shorter chain showed no activity. The C17-epimer **25** lacked significant HDAC inhibitory activity compared with **1**, suggesting that the four-atom linker between the macrocycle and the octanoyl group in the side chain and the (*S*)-configuration at the C17-position are all critical to the potent HDAC inhibitory activity of **1**. The valine residue in the macrocycle can be replaced with the alanine without compromising its activity to a large extent.

**Scheme 5:**
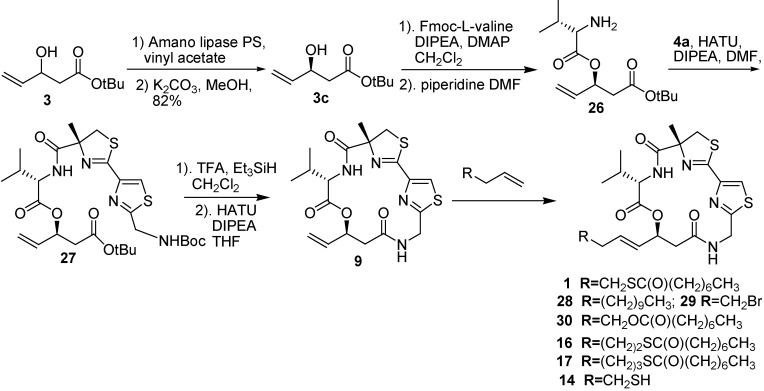
Synthetic route of largazole and its analogues developed by Cramer and co-workers.

Cramer and co-workers [7] have developed a short route for the synthesis of largazole (**1**), in which largazole was obtained in 19% overall yield. The β-hydroxy ester fragment **3c**, which was obtained by an enzymatic resolution of racemic alcohol, was subjected to the esterification with Fmoc-L-valine followed by the deprotection of the Fmoc group to provide **26**. Nitrile **5** was allowed to react with (*R*)-α-methylcysteine hydrochloride under mild aqueous conditions to provide the thiazoline acid **4a** in an excellent yield. The intermediate **27** was prepared by coupling **4a** with the amine **26** in the presence of HATU. The deprotection of **27** provided the lactam **9** in 77%–89% yield in a dilute solution of HATU and Hüing’s base. In the optimization of the cross-metathesis reaction, Cramer and co-worker found a yield increased from 44% to 75% when the *p*-nitro-substituted catalyst was used instead of the Hoveyda-Grubbs second-generation catalyst with the catalyst loading of 15% in CH_2_Cl_2_ at 80–100 °C. Thus, Largazole and its analogues **14**, **16**, **17**, **28**, **29**, and **30** were prepared in comparable yields (37%–92%) under the optimized conditions (Scheme 5).

The findings of the antiproliferative activity of **1** and its analogues showed that the octanoic thioester of **1** acts as a protecting group and that the free thiol **14** has slightly lower potency but significantly higher specificity compared with the thioester of **1**. The intermediate **9** and the analogue **28** showed no growth inhibitory activity. The replacement of the thioester with an ester group in the compound **30** led to a complete loss of activity. Thioester derivatives **16** and **17** showed no activity at all. It suggests the importance of positioning thio functionality at the right distance from the cyclic core and the necessity of the thiobutenyl group for its antiproliferative activity.

**Scheme 6:**
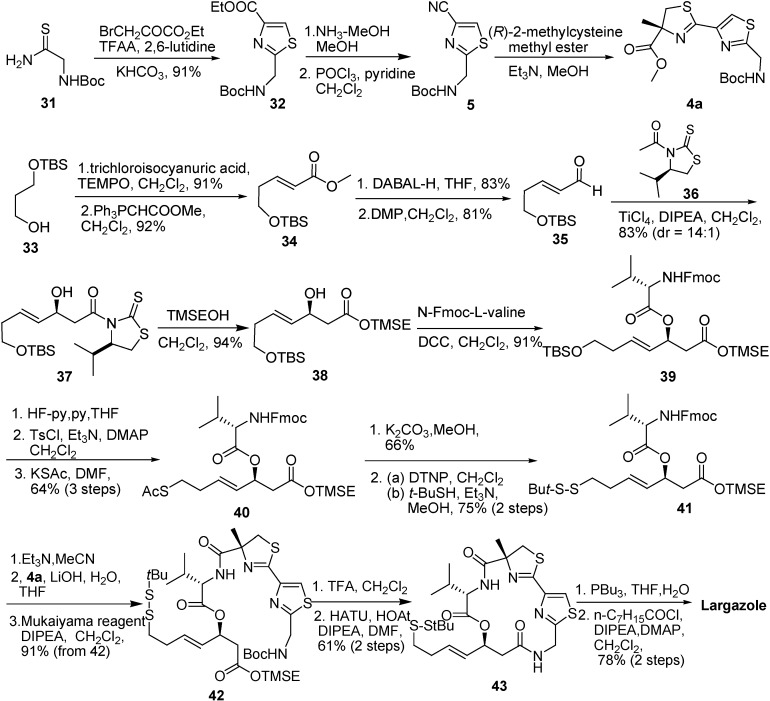
Synthetic route of largazole developed by Ye and co-workers.

Ye and co-workers [8] have accomplished the total synthesis of **1** in 5.8% overall yield from 3-[(*tert*-butyldimethylsilyl) oxy] propane **33**. The allylic alcohol fragment **37** was prepared by a series of reactions: oxidation of alcohol **33**, Wittig reaction, reduction of ester **34**, oxidation of **34** to give the enal **35**, and then reaction with Nagao’s chiral *N*-acetylthiazolidine-2-thione **36** [4,5]. The displacement of the auxiliary of the alcohol **37**, followed by the esterification of **39**, provided the intermediate **40**, which was converted to its corresponding disulfide **41**. The fragment **4a** was prepared according to Pattenden’s procedure (Et_3_N, MeOH, 50 °C) [9]. Hydrolysis of the methyl ester **4a** provided the acid, and it was coupled with the product obtained from the removal of the Fmoc group of the disulfide **41** to give the linear depsipeptide **42**. The deprotection of both Boc and TMSE ester groups of the depsipeptide **42** followed by treatment with HATU provided the cyclodepsipeptide **43**. Largazole was prepared by the reductive cleavage of the disulfide bond in the cyclodepsipeptide **43** followed by the reaction with octanoyl chloride (Scheme 6).

**Scheme 7:**
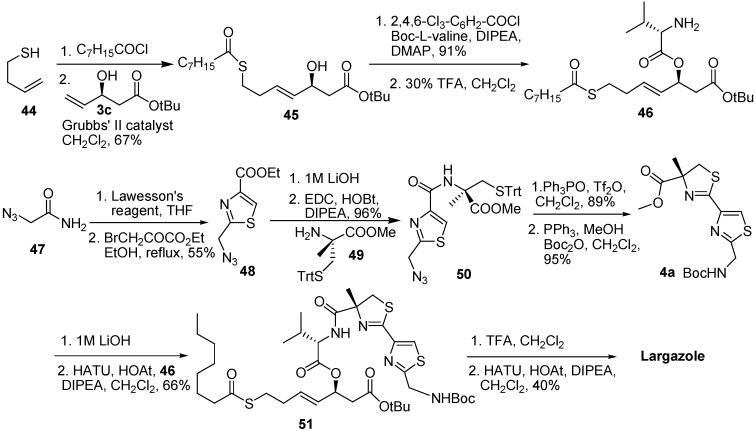
Synthetic route of largazole developed by Ghosh and Kulkarni.

Ghosh and Kulkarni have also completed an enantioselective total synthesis of largazole [10]. The condensation of the thiazole acid **48** and protected (*R*)-2-methylcysteine (**49**) gave **50**, and then treatment of **50** according to Kelly’s procedure [11] followed by reduction and protection provided the thiazole ester **4a**. The requisite thioester was obtained by reaction of 3-butene-1-thiol (**44**) with octanoyl chloride. A cross-metathesis of thioester and alcohol **3c** was performed followed the same way used by Cramer and his co-workers in the presence of 3 mol % of Grubbs’ second-generation catalyst. The Yamaguchi esterification of *N*-Boc-valine, the deprotection of the Boc group, and the saponification of the thiazole ester **4a** were performed. The coupling of the amine **46** to the acid **4a** was carried out by using HATU and HOAt to prepare **51**. By removing the protecting Boc and *tert*-butyl groups of **51**, compound **1** was synthesized under dilute conditions with HATU (2 equiv.) and HOAt (2 equiv.) in the presence of diisopropylethylamine (Scheme 7).

**Scheme 8:**
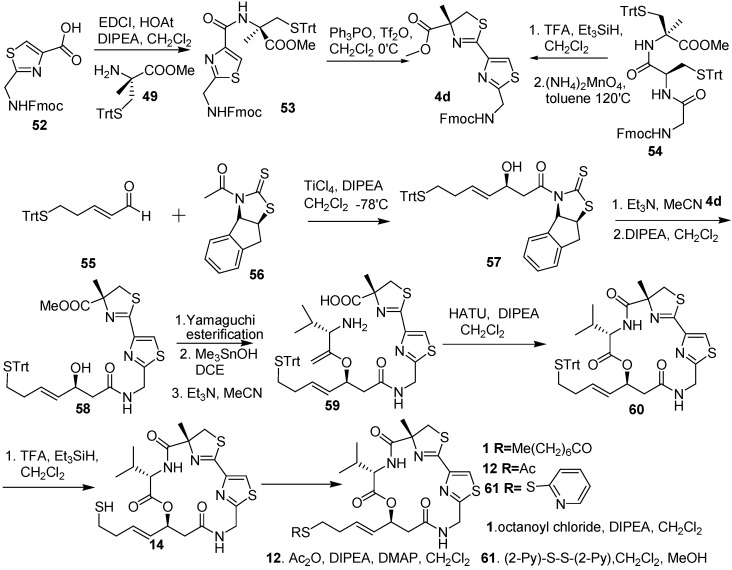
Synthetic route of largazole and its analogous by Doi and co-workers.

Doi and co-workers [12] synthesized unit **4d** using Kelly’s method, as did Ghosh and co-workers. They also carried out one-pot bisthiazoline formation using Ishihara’s method [13]. Unit **57** was prepared by the asymmetric aldol reaction of aldehyde **55** and a modified Nagao reagent, *N*-acetyl-thiazolidinethione **56** [14]. The subsequent amidation of **57** with the amine prepared from **4d** by the removal of the Fmoc group provided a thiazoline–thiazole alcohol **58**. To synthesize **59**, the thiazoline–thiazole alcohol **58** was subjected to the Yamaguchi esterification of the hydroxyl group with Fmoc-Val-OH, hydrolysis of methyl ester, and then removal of the Fmoc group. The desired cyclic depsipeptide **67** was obtained by using HATU and DIPEA under high-dilution conditions. The deprotection of the trityl group of **60** provided thiol **14**, and then it was allowed to react with octanoyl chloride to give **1**. The analogous **12** and **61** were prepared by treating the thiol **14** with acetic anhydride and 2ʹ2-dipyridyl disulfide, respectively (Scheme 8).

The HDAC inhibitory activity of Largazole and its analogous **12**, **14**, and **61** was similar, whereas the thiol **14** was slightly more potent than the *S*-substituted compounds, suggesting that thiol **14** is probably a real active form that is released within the cells.

**Scheme 9:**
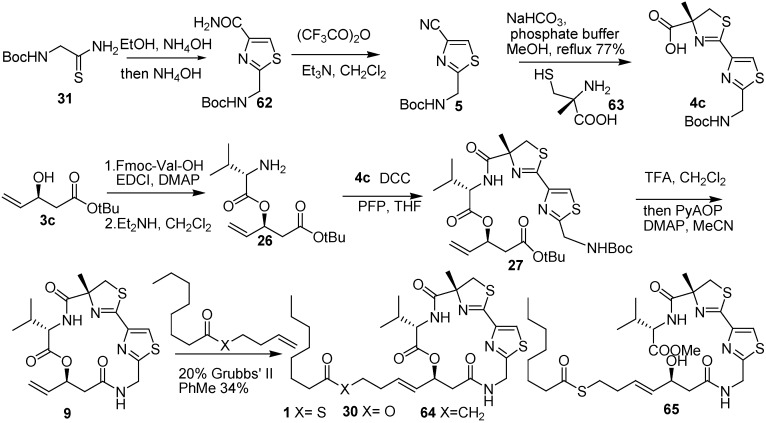
Synthetic route of largazole and some analogues developed by Phillips and co-workers.

Phillips [15] and co-workers synthesized the macrocycle **9** started with the Boc-protected glycine thioamide **31** and ethyl α-bromopyruvate converted to thiazolyl amide **62** by Hantzsch thiazole synthesis [16]. Dehydration of amide **62** produced the nitrile **5**, and it was allowed to react with α-methylcysteine to give thiazolylthiazoline **4c** under mild aqueous conditions, the same as Cramer and his co-workers. Coupling of Fmoc-Val-OH with the β-hydroxy ester **3c**, which was obtained by enzymatic resolution of its corresponding racemic aldol adduct, and the subsequent removal of the Fmoc group provided amine **26**. Condensation of amide **26** and **4c** was accomplished with DCC and pentafluorophenol. After removal of Boc and *tert*-butyl groups, **27** was subjected to macrolactamization with PyAOP and DMAP in acetonitrile to provide macrocycle **9** in 50% yield. **1** was obtained in 34% yield in the presence of 20 mol% of Grubbs’ second-generation catalyst (Scheme 9).

The prepared analogues such as ester **30**, ketone **64**, seco-ester **65**, macrocycle **9,** and largazole (**1**) were tested for differential inhibitory activity. The results showed that only largazole preferentially acted on tumor cells. It is shown that the cellular target of largazole is HDAC and that the conformation of the depsipeptide is important for targeting HDAC, as **9**, **30**, **64**, and **65** did not show any activity. Phillips and co-workers also confirmed the preliminary conformation of largazole.

**Scheme 10:**
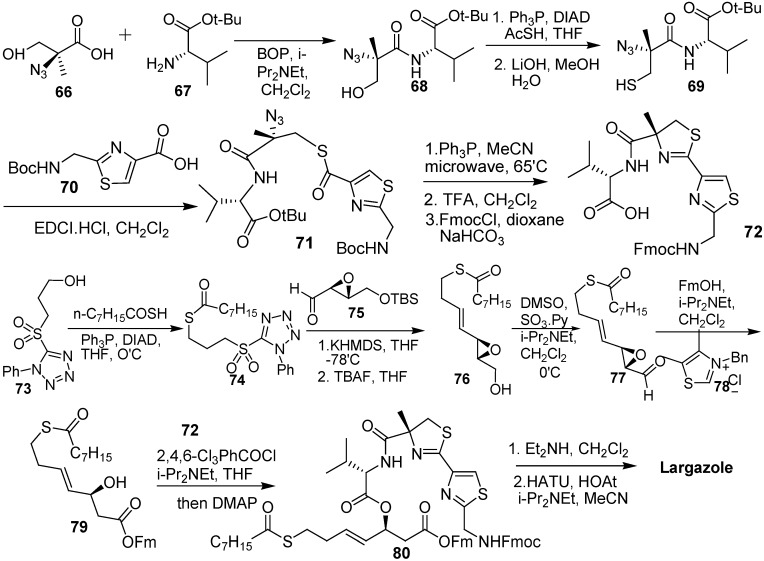
Synthetic route of largazole developed by Forsyth and Wang.

Forsyth and Wang [17] developed a novel synthesis strategy. The Julia olefination using α,β-epoxy aldehyde **75** and thioester **74** containing a tetrazolyl sulfone moiety, which was prepared by the Mitsunobu reaction, gave alkenes. Removal of the TBS protecting group and the oxidation of the primary alcohol of **76** provided the unstable α, β-epoxy aldehyde **77**. The requisite **79** was prepared from α,β-epoxy aldehyde **77** by *N*-heterocyclic carbene–mediated esterification with fluoren-9-ylmethanol. The azido thioester **71** was prepared from **66** by a series of reactions: amide formation, thiol substitution, and thioesterification. Treatment of the azido thioester **71** with PPh_3_ in acetonitrile under microwave irradiation gave the polypeptide. The acid fragment **72** was obtained by the deprotection of Boc and *tert*-butyl groups of the polypeptide and by an efficient Fmoc derivatization. Largazole was obtained by the Yamaguchi esterification of **79** and acid **72**, followed by deprotection and macrolactamization (Scheme 10).

**Scheme 11:**
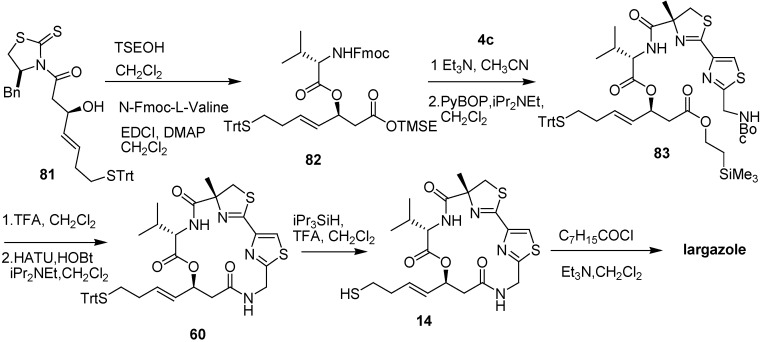
Synthesis of largazole developed by Williams and co-workers.

Williams and co-workers [18] synthesized the thiazoline–thiazole subunit **4c** by condensation of nitrile **5** and (*R*)-2-methylcysteine methyl ester hydrochloride under basic conditions (triethylamine in methanol, Scheme 3). Thiazolidinethione **81** was treated with 2-(trimethylsilyl)ethanol to prepare TSE-protected acid, and then it was allowed to couple with *N*-Fmoc-L-Val to prepare **82**. The deprotection of the Fmoc group of **82**, followed by PyBOP-mediated coupling with the thiazoline–thiazole subunit **4c** provided **83**. Removal of protecting Boc and TMSE groups of **83** under high dilution in the presence of HATU (2 equiv) and HOBt (2 equiv) gave the macrolactamization product **60**. Removal of the protecting *S*-trityl group from **60** provided the thiol **14**. The acylation of **14** with octanoyl chloride under standard conditions produced largazole (Scheme 11).

**Figure 2:**
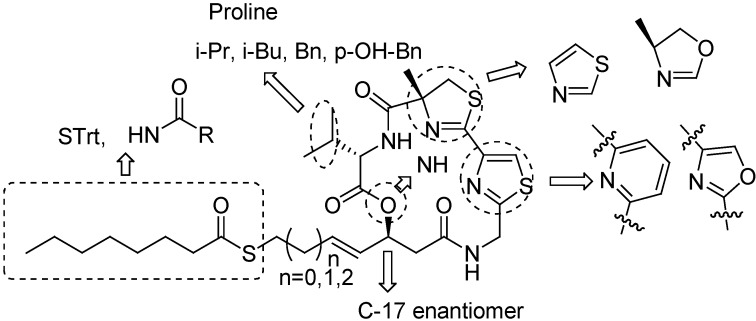
The structural modification pathways of largazole.

During the synthesis of largazole, a wide range of analogues were obtained by Williams and co-workers [19]. According to the assay data of these analogues (Figure 2), the potency of the enantiomer of largazole decreased by almost three orders of magnitude. The C-2 epimer, the valine-to-proline substitution, and the thiazole–thiazole derivative showed intermediate potency. It shows the importance of the obligate stereochemical and conformation activity relationships between the natural product and its protein targets. The oxazoline–oxazole analogue showed a similar activity compared with largazole. It suggests that the role of the natural 3-hydroxy-7-mercaptohept4-enoic acid moiety in Largazole is significant for its activity, which can also be known in FK228 and spiruchostatin. The thiazole–pyridine substitution possessed subnanomolar activity, which was three to four times more potent than largazole [20]. It seems that the methyl substituent of the thiazoline ring is not essential for the potency of the natural product.

**Scheme 12:**
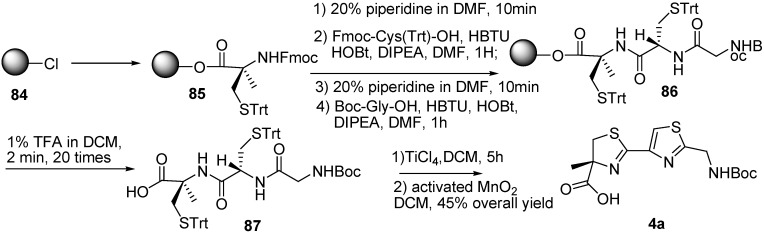
Synthesis of thiazoline–thiazole fragment **4a**.

Recently, our efforts have been focused on the total synthesis of largazole [21], we performed the solid-phase synthesis of key fragment byusing the 2-chlorotrityl chloride resin **84**. The thiazoline–thiazole fragment **4a** was successfully prepared by tandem deprotection–cyclodehydration and subsequent oxidation with activated manganese dioxide (Scheme 12).

**Scheme 13:**
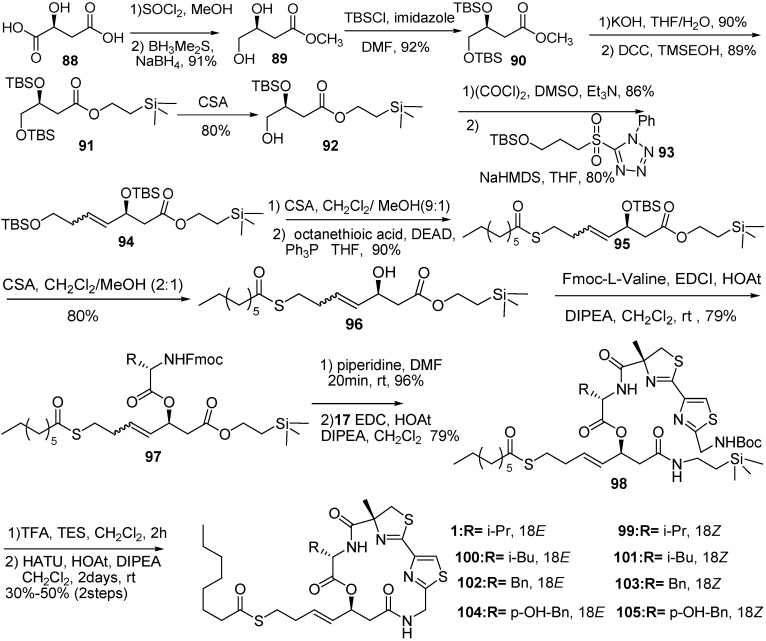
Synthesis of largazole developed by our group.

The primary alcohol **92** was prepared from commercially available (−)−malic acid (**88**). Swern oxidation of the alcohol and the Julia–Kocienski olefination followed by coupling with sulfone gave the key intermediate **94** in a favorable *E/Z* ratio (8/1). The selective deprotection of the primary TBS and the Mitsunobu reaction provided **95**. The allylic alcohol was coupled with enantiomerically pure amino acids to prepare the intermediate **97**. Removal of the Fmoc group followed by coupling with thiazoline–thiazole acid **4a** gave cyclization precursor **98**. The 16-member cycloamide was prepared by removal of protected group and subsequent macrolactamization (Scheme 13).

Using this strategy, a small library of largazole analogs was developed. The analogues with *cis*- alkenes showed no activity against either human tumor cell lines or normal cell lines. Compound **104** showed slightly lower potency, but much improved selectivity for cancer cell lines. Structure-activity relationships studies suggested that the geometry of the alkene in the side chain is critical. While the largazole analogues with *trans*-alkene are potent for the antiproliferative effect, those with *cis*-alkene moieties are completely inactive. Most importantly, replacement of valine with tyrosine in largazole increased selectivity toward human cancer cells over human normal cells more than 100-fold.

## 3. Conclusions

The recent progresses in synthesis of largazole and its analogues are reviewed, in which several competitive synthetic routes, and its preliminary structure–activity relationships are discussed. Studies on the biological evaluation of largazole and its analogues have revealed that largazole is a pro-drug, which inhibits the growth of cells by releasing active free thiol group within the target site. A large body of investigations has provided important insight into the structural, functional, stereochemical, and conformational aspects of the largazole molecular scaffold which will constitute the basis for the further design and synthesis of extraordinarily potent HDAC inhibitors with potential therapeutic significance.

## Abbreviations

BOP: (1*H*-benzotriazol-1-yloxy) tris(dimethylamino)-phosphonium hexafluorophosphate
DCC: dicyclohexylcarbodiimide
DIAD: diisopropyl azodicarboxylate
DMAP: 4-(dimethylamino)pyridine
DMF: dimethylformamide
DMSO: dimethylsulfoxide
EDC: 1-ethyl-3-(3’-(dimethylamino)propyl)carbodiimide
HATU: -[(dimethylamino)-1*H*-1,2,3-triazolo[4,5-b]pyridin-1-ylmethylene)-*N-*methyl-methanaminium hexafluorophosphate *N*-oxide
HBTU: *N*-[(1*H*-benzotriazol-1-yl)-(dimethylamino)methylene]- *N-*methylmethanaminium hexafluorophosphate *N*-oxide
HOAt: 1-hydroxy-7-azabenzotriazole
HOBt: 1-hydroxybenzotriazole
TBAF: tetrabutylammonium fluoride
TEMPO: 2,2,6,6-tetramethylpiperidine 1-oxyl
TFA: trifluoroacetate
THF: tetrahydrofuran
